# A Trickster in Disguise: Hyaluronan’s Ambivalent Roles in the Matrix

**DOI:** 10.3389/fonc.2017.00242

**Published:** 2017-10-09

**Authors:** Lena Bohaumilitzky, Ann-Kathrin Huber, Eva Maria Stork, Simon Wengert, Franziska Woelfl, Heike Boehm

**Affiliations:** ^1^Institute of Pharmacy and Molecular Biotechnology, University of Heidelberg, Heidelberg, Germany; ^2^CSF Biomaterials, Max Planck Institute for Medical Research, Heidelberg, Germany; ^3^Department of Biophysical Chemistry, University of Heidelberg, Heidelberg, Germany

**Keywords:** hyaluronan, naked mole rat, cancer, cancer resistance, early contact inhibition, aging, cellular senescence, CD44

## Abstract

Hyaluronan (HA) is a simple but diverse glycosaminoglycan. It plays a major role in aging, cellular senescence, cancer, and tissue homeostasis. In which way HA affects the surrounding tissues greatly depends on the molecular weight of HA. Whereas high molecular weight HA is associated with homeostasis and protective effects, HA fragments tend to be linked to the pathologic state. Furthermore, the interaction of HA with its binding partners, the hyaladherins, such as CD44, is essential for sustaining tissue integrity and is likewise related to cancer. The naked mole rat, a rodent species, possesses a special form of very high molecular weight (vHMW) HA, which is associated with the extraordinary cancer resistance and longevity of those animals. This review addresses HA and its diverse facets: from HA synthesis to degradation, from oligomeric HA to vHMW-HA and from its beneficial properties to the involvement in pathologies. We further discuss the functions of HA in the naked mole rat and compare them to human conditions. Though intensively researched, this simple polymer bears some secrets that may hold the key for a better understanding of cellular processes and the development of diseases, such as cancer.

## Prologue

This is the story of a young researcher whose child became ill with cancer. So far, all therapeutic trials have failed and always the cancer relapsed. The months passed and now, we are writing the year 2017. The sun was just rising above the horizon as our researcher woke up with a startled expression. Although he could not remember his dream of the night before, there were still two pictures which he could not get out of his head. What is it between the naked mole rat and its extraordinarily long hyaluronan (HMW-HA)? Was this a sign of destiny showing him a way to save his child? Pondering this question, he went to his laptop and opened a search. The number of returned results deflated him, but yes, there was a connection between the sugar molecule and the exceptional rodent. Driven by eager anticipation, a journey through scientific publications, data and knowledge began…

## HA—Simple but Diverse

**Figure 1:**
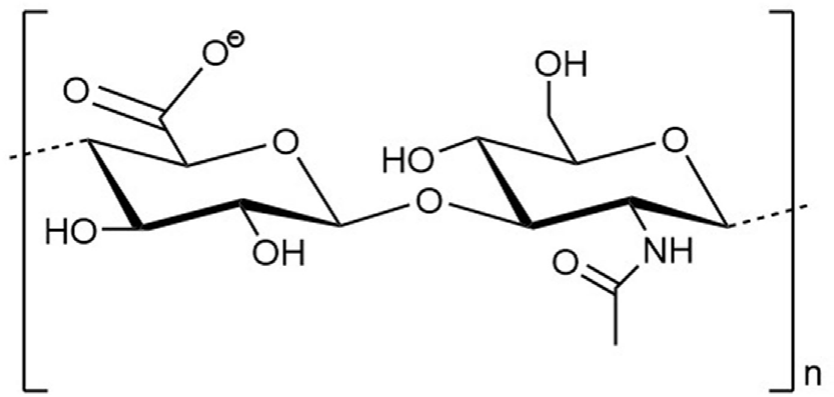
Structural formula of a disaccharide building block of the hyaluronan polymer composed of alternating d-glucuronic acid and *N*-acetyl-d-glucosamine units. *n* indicates the number of repeating units in a polymer molecule.

Hyaluronan is a polysaccharide that is characterized by a simple chemical structure but has extraordinary biological properties (1). As a key component of the vertebrate extracellular matrix (ECM), the linear biopolymer is composed of alternating d-glucuronic acid and *N*-acetyl-d-glucosamine units, connected *via* β-1,3- and β-1,4-glycosidic bonds (Figure 1) (2–4). Under normal physiological conditions, HA consists of 2,500–17,500 U with a molecular weight of 1,000–7,000 kDa (5). A single HA polysaccharide can thus reach a polymer length of 2.5–17.5 μm. The repeating sequence is conserved in all vertebrates and except for occasional deacetylated glucosamine residues, not modified in its chemical structure. At physiological pH values, the carboxyl group of each d-glucuronic acid unit is usually dissociated, which results in the formation of a negatively charged biomolecule (3, 6, 7).

Hyaluronan belongs to the family of glycosaminoglycans (GAGs). However, in contrast to its other family members, such as heparin sulfate and chondroitin sulfate, HA is synthesized as an unmodified, non-sulfated polysaccharide which is directly extruded into the ECM (8). Within the ECM, HA constitutes an extracellular scaffold that coordinates the attachment of other ECM components, such as proteoglycans (9, 10). Apart from the interaction with the heavy chains of the serum protein inter-α-inhibitor, which is mediated *via* a direct ester bond (11), the linkage of HA to other HA-binding proteins is achieved in a non-covalent manner (10, 12). These proteins, also termed hyaladherins, comprise cell surface receptors, as well as ECM and blood plasma proteins (13, 14).

Hyaluronan-binding results in a variety of intracellular as well as extracellular responses. The interactions of HA with cell surface receptors induce numerous intracellular signaling pathways, for example, those regulating proliferation or cell motility (15). The often multivalent interactions of HA with ECM proteins support the generation of huge HA-organized extracellular matrices and, thus, help to provide the structural integrity of many tissues (16–18).

At a low level, HA is expressed ubiquitously in the human body (19). It is proposed that adult humans contain about 12–15 g of HA, the majority of which (more than 50%) occurs in the skin (20). Furthermore, it is found in connective tissue, synovial fluid, intervertebral disks, and the vitreous body of the eye (2).

Hyaluronan synthesis is also strictly regulated in embryonic development (21). HA constitutes a main component of fetal tissues, fetal structures, such as the Wharton’s Jelly of the umbilical cord, and the amniotic fluid (22). It also plays an important role in condensation events and in epithelial to mesenchymal transition (18, 23).

Besides, HA shows remarkable physical and biological properties. HA is highly hygroscopic, tightly binding 15 water molecules with each disaccharide unit (24). Thus, HA has a great ability to retain water, for example, 1 g of HA might retain 6 l of water (25). Furthermore, HA shows a very high and shear-dependent viscoelasticity, resulting in the role of HA as an extracellular lubricant (26). As a consequence of these remarkable hydrodynamic properties in terms of water retention and viscosity, HA is essential to maintain tissue hydration, tension, and integrity (3).

The molecular weight of HA varies and has great impact on its physiological functions and activities (27). Above 1,000 kDa HA is defined as high molecular weight HA (HMW-HA). HMW-HA possesses anti-inflammatory, anti-proliferative, and anti-angiogenic properties and is, furthermore, involved in wound healing processes (5, 27, 28). In homeostasis, HA is found in its HMW form in almost all human tissues. However, pathological circumstances, such as inflammation, show evidence for an elevated HA fragmentation resulting in a higher level of HA polymers with a lower molecular weight (29). Therefore, the effects of HA in the pathological context are often associated with the variable mass of the polymer (5).

Interestingly, the available molecular weight range of HA in different organisms is not consistent. A unique very high molecular weight (vHMW) HA can be found in the naked mole rat (30). A comparison of human and naked mole rat HA is of interest because recent findings provide evidence for a link between the naked mole rat’s cancer resistance and its extremely HMW-HA.

## The Naked Mole Rat—An Extraordinary Rodent

**Figure 2:**
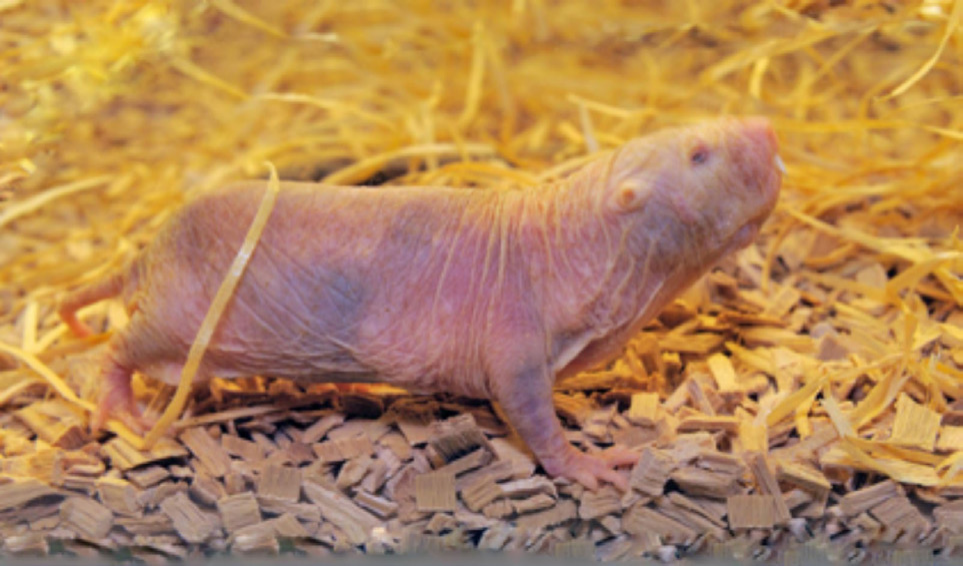
Naked mole rat in captivity (© Tiergarten Schoenbrunn, Austria/Norbert Potensky).

The naked mole rat is a hairless, mouse-sized rodent (Figure 2) that inhabits subterranean arid regions in northeast Africa, mainly Kenya, Ethiopia, and Somalia (31). Naked mole rats exhibit an abundance of unusual characteristics, such as eusociality [reviewed in Ref. (32)], pain insensitivity [reviewed in Ref. (33)], and poikilothermic thermoregulation [reviewed in Ref. (34)]. But even more fascinating, naked mole rats are the longest living rodents with a lifespan of up to 30 years (35) and show a high cancer resistance (30).

Naked mole rats show peculiar features regarding the molecular weight and distribution of HA. Compared to other species, naked mole rats exhibit HA enrichment in kidney, brain, heart, and skin. These elevated HA levels derive from altered enzyme activities (30). While the HA synthases 1 and 3 (HAS1/3) in naked mole rats show expression levels similar to mice and humans, an increased expression of HA synthase 2 (HAS2) can be observed in naked mole rats. In addition, their low levels of Hyal lead to a slower degradation of HA (30, 36). Moreover, naked mole rat HA has a vHMW, between 6 and 12 MDa (30, 37). The elevated amount of HA and its unusually high molecular weight might be caused by an amino acid alteration in the active site of HAS2. In this site, two asparagines are substituted by two serines, which is unique for the naked mole rat since these amino acids are highly conserved among all other mammals (30). However, if human cells are transfected with cDNA of the mutated naked mole rat’s HAS2, these cells also produce vHMW-HA (30). This clearly indicates that this small alteration of HAS2 is causing the increased size of HA in naked mole rats (30). Since the resulting vHMW-HA has been linked to different aspects of the naked mole rat’s unique properties, it might be of interest for understanding the role of HA in other organisms.

### Cancer Resistance of Naked Mole Rats

Hypersensitivity to contact inhibition is one of the unique properties of naked mole rats: they show the so-called early contact inhibition (ECI). Thus, naked mole rat cells arrest cell proliferation when only few cell–cell contacts are formed and never reach the same densities as human or murine cells (38).

Contact inhibition by itself is a powerful anticancer mechanism and causes an arrest of the cell cycle when cells contact each other. As a consequence, the formation of multilayers and uncontrolled growth is prevented, which is not true for cancer cells since they have lost this ability (38). The contact-induced growth arrest is mainly mediated by the cyclin-dependent kinase inhibitors p27^Kip1^ (p27) and supported by p16^INK4a^ (p16) (38).

**Figure 3:**
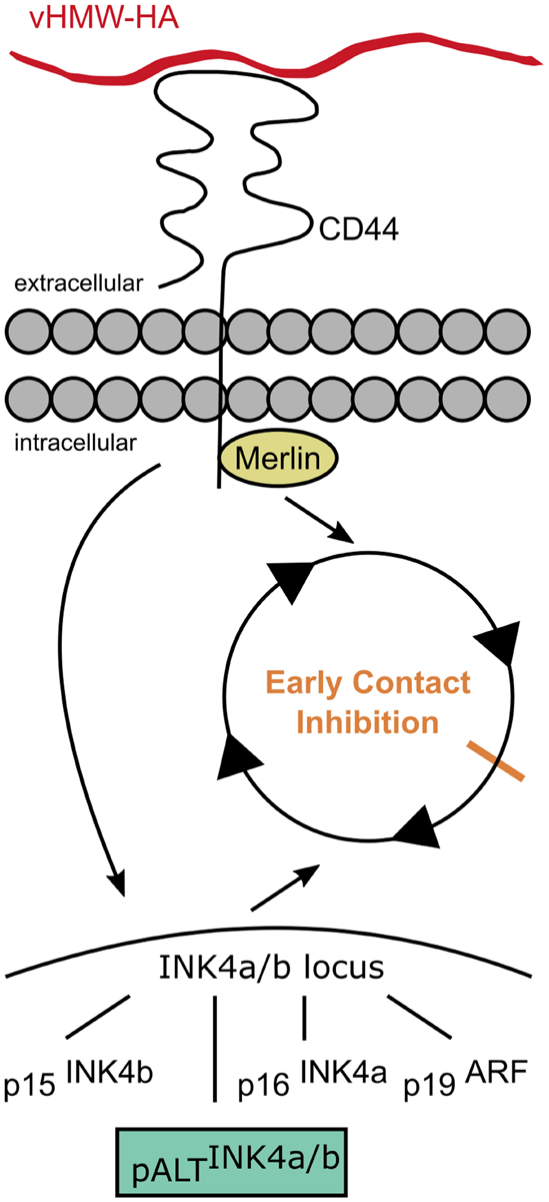
Early contact inhibition. The uniquely mutated hyaluronan synthase 2 of the naked mole rat produces very high molecular weight HA (vHMW-HA) which triggers a signaling cascade *via* the CD44 receptor activating the INK4a/b locus and inducing the intracellular interaction of CD44 with merlin. The activated INK4a/b locus encodes for the tumor suppressors p15, p16, and p19 that arrest the cell cycle. In addition, naked mole rats possess a unique fourth hybrid form, pALT, which stops the cell cycle more efficiently. Interactions of CD44 with merlin contribute to the cell–cell contact-induced growth arrest.

The ECI is linked to the described cancer resistance of naked mole rats (39). Liang et al. showed that naked mole rat fibroblasts are resistant to experimental oncogenic transformation with Ras^G12V^ and SV40 large T antigen, unlike other mammalian cells (40, 41). ECI is triggered by vHMW-HA and its interaction with the CD44 receptor (39). Moreover, the cytoplasmic side of the CD44 receptor interacts with merlin (neurofibromin 2), which regulates contact inhibition (Figure 3) (30). It has been shown that naked mole rat fibroblasts that were cultured with bacterial hyaluronidase grew completely confluent and lost the ECI-phenotype due to the lacking trigger in the form of vHMW-HA (30). Similar results were obtained when the CD44 receptor was blocked with antibodies (30).

Furthermore, the interaction of vHMW-HA with the CD44 receptor activates a signaling cascade, which causes the induction of the INK4a/b locus (42). The INK4a/b locus plays an important role in cancer development and encodes for three different mammalian tumor suppressors: the cyclin-dependent kinase inhibitors p15^INK4b^ (p15) and p16^INK4a^ (p16) as well as p19^ARF^ (p19), a repressor of the MDM2 oncogene. Out of those tumor suppressors, the level of p16 is elevated in naked mole rat cells that exhibit ECI (38, 42). While the contact inhibition in humans or mice is primarily mediated by the p27 cyclin-dependent kinase inhibitor; in naked mole rats, this suppressor seems to only serve as a backup if the ECI is not functional (38).

In addition to the upregulation of p16, a fourth unique product of the INK4a/b locus has been found in naked mole rats. This isoform, pALT^INK4a/b^, is capable of arresting the cell cycle more efficiently than the other INK4a/b suppressors and does so independently of HA–CD44 interactions (42).

The lack of described neoplasia cases in naked mole rats have led to the assumption that this species is essentially cancer-free, but recently two cases of cancer in naked mole rats were reported. Two naked mole rats from different US zoological institutions are the first described cases of cancer in naked mole rats. In addition, pre-cancerous lesions were also found in several other naked mole rats (43). These findings illustrate that naked mole rats may develop cancer and raise further questions about cancer resistance mechanisms and their scope. Detailed investigation of the causes and documentation of such rare cases will benefit the current cancer research and increase recent knowledge concerning cancer resistance mechanisms.

### Longevity of Naked Mole Rats

The lifespan of mammals is usually linked to their average body mass. In general, a doubling of body mass leads to a 13% increase in lifespan (44). However, the small naked mole rats are exceeding this rule up to ninefold with a maximum lifespan of approximately 30 years (44). High cancer resistance, eusocial behavior, and a protected subterranean habitat contribute to the long lifespan of naked mole rats (45).

In addition to the extraordinary lifespan, the aging process in itself differs from every other mammal. Aging or senescence has been described as the progressive loss of tissue and organ function over time (46). Generally, aging is associated with an increased mortality risk, declining fertility and a functional degradation and occurs in every organism, except for certain cold water fish and long-lived trees (44). The term “negligible senescence” refers to a lack of age-related changes concerning reproductive and physiological functions and was coined by Caleb Finch to describe slow aging species (44). Finch defined three criteria to determine if an organism exhibits negligible senescence: (i) decreasing mortality rate, (ii) consistent physiological functions except for reproduction, and (iii) constant reproduction rate over lifetime (47). Mortality rates of naked mole rats show a decrease with increasing age as they are most likely to die in the nursing period due to colony neglect, lack of maternal care, cannibalism, or starvation (31, 44). Physiological functions, such as basal metabolic rate, arterial elasticity, and bone mineral content, show no changes up to an age of 25 years and are, therefore, compliant with the defined terms (44, 47). No age-related change in litter size could be observed although the survival rate of pups decreases with increasing age. Thus, the second criterion of negligible senescence does not fully coincide with the defined characteristics. Nevertheless, naked mole rats fulfill almost all criteria of negligible senescence (47). The overall maintained good health of naked mole rats far into their third decade of life is equivalent to an 80-year-old human with the health status of a 30-year-old (40).

It has been proposed that aging-related mechanisms are similar to those that mediate stress resistance. Thus, the long lifespan of the naked mole rat could be correlated with high stress resistance (48). Compared to murine cells, naked mole rat cells are more resistant to stressors, such as cadmium, methyl methanesulfonate (DNA alkylating agent), paraquat (oxidative stress inducing agent), heat, and low glucose media, consistent with the initial hypothesis. Interestingly, naked mole rat cells are also more sensitive to H_2_O_2_, UV light, and rotenone (mitochondrial inhibitor) in comparison to murine cells (48).

Lewis et al. further investigated stress resistance in naked mole rat cells by extending experiments with different cytotoxins and adjusting culture conditions. Fibroblasts derived from naked mole rats showed a higher resistance to different stresses, including heat, heavy metals, xenobiotics, and DNA-damaging agents, compared to cells derived from mice. The determined LD50 (median lethal dose) values varied between 2- and 20-fold increase in naked mole rat fibroblasts. These findings support the link between stress resistance and longevity. Furthermore, naked mole rat cells show a prolonged cell cycle arrest and stop proliferation at a low toxin concentration (40).

The resistance to oxidative stress in naked mole rats and the possible link to their longevity were studied separately since the “oxidative stress theory” is only one approach to explain aging (49). Naked mole rats surprisingly show similar levels of reactive oxygen species (ROS) as short-lived species such as mice and the antioxidant defense is not significantly different (44). Compared to mice, naked mole rats show a 70-times lower activity of cellular glutathione peroxidase but higher activities of several other antioxidant enzymes, such as manganese superoxide dismutase. Furthermore, antioxidant activity undergoes no age-related changes in naked mole rats, however, such activity can be detected in mice. These findings indicate that oxidative stress resistance is not the key player in negligible senescence and longevity of naked mole rats (48). However, the connection between stress resistance, the long-lasting health, and longevity cannot be denied and further research is required.

Due to its unique anticancer mechanism, negligible senescence, and unusually long lifespan, the naked mole rat serves as an ideal model for studies on aging and cancer.

## HA in Aging and Cellular Senescence

The link between HA and aging, as prominent in the naked mole rat, can also be found in other species. In contrast to aging, sometimes also referred to as senescence, cellular senescence describes a cell cycle arrest and is not necessarily linked to the aging process. Cellular senescence is involved in tissue repair and age-related diseases, but can also act as a potent anticancer mechanism by causing a cell cycle arrest in tumor cells (50).

Aging correlates with a decrease of HA content in the human body (51). If that also holds true for naked mole rats has to be investigated. Normally, the HA level rapidly increases during early development, followed by a continuous decrease over lifetime. For instance, the HA content in the basal and spinous layers of the epidermis was found to be reduced significantly 4 weeks after birth and to be as low as in adult phenotypes 2 months after birth in mice (52). Many processes occurring during a lifetime contribute to this decline in HA content. For example, chronic UVB irradiation declines the amount of HA in the dermis *via* inhibition of HA synthesis (53).

In general, downregulation of the hyaluronan synthases (HAS) enzymes, particularly HAS2, seems to be linked with cellular senescence and with aging. For instance, microRNA-23a-3p was discovered to downregulate expression of HAS2 in human fibroblasts, leading to significantly decreased amounts of extracellular HA. Since the fibroblasts in this study were taken from young and old donors, the increased level of microRNA-23a-3p could be associated not only with cellular senescence but also with aging (54). If a similar regulatory mechanism based on microRNA also applies in naked mole rats, it has not been investigated so far and demands further study.

Furthermore, senescent human mesenchymal stem cells (MSCs) express significantly lower amounts of the vascular cell adhesion molecule 1 (VCAM-1), which is important for the wound healing effect of MSCs. Interestingly, the expression of VCAM-1 could be recovered either by adding HA or by crosslinking CD44, thereby mimicking CD44 clustering by HA binding (55). Taking together the findings that HAS2 is downregulated in senescent MSCs and that compensating that loss with HA leads to recovery of VCAM-1, it was concluded that the interaction of HA with its receptor CD44 is correlated with the expression of VCAM-1 in senescent MSCs (55). Bearing in mind that cellular senescence is associated with aging, HA may influence cell adhesion and migration in the elderly.

In addition, the interplay of HA and its binding proteins, the hyaladherins, is crucial to cellular senescence. Generally speaking, the interaction of HA and versican is important for forming the ECM (56). The loss of interactions between HA and versican was found to induce cellular senescence in murine cell lines (57). The authors of the study generated knock-in mice, in which the HA-binding domain for versican showed a reduced binding affinity for HA. Consequently, unbound HA was fragmented using a hyaluronidase. By blocking the extracellular membrane receptor CD44 with an antibody, these HA fragments were confirmed to bind to CD44 by detecting a decrease in CD44 signaling. Suwan et al. further could show that the phosphorylation of extracellular signal-regulated kinase (ERK) 1/2, a known downstream signaling pathway of CD44, was increased significantly by treatment with HA fragments, leading to cellular senescence in those knock-in mice, respectively. Accordingly, fragmentation of HA has been associated with an increase in cellular senescence (57).

So far it has been shown that downregulation of HAS2 and, therefore, a lack of HMW-HA contributes to undesirable cellular senescence. On the other side, cellular senescence could eventually be used to arrest altered cells in their cell cycle, preventing further mitosis and thereby further spread of the disease. For example, induced cellular senescence was proposed as a therapeutic strategy against fibrosis (58). Fibrosis is a complex disease with heterogeneous phenotypes and chronic fibroproliferative diseases are involved in approximately 45% of all deaths in the so-called developed world (59). It correlates with chronic inflammation and intense accumulation of a rigid ECM. Thereby, the otherwise flexible binding tissue is replaced by a scar-like stiff tissue leading to a loss of mechanical integrity in affected tissues. In this way, affected patients can suffer from organ malfunctions which can also be lethal (60). Though the pathology of fibrosis is not yet fully understood, it can be regarded as a continuous tissue repair response that goes hand in hand with fibroblast-to-myofibroblast transition (60).

In this context, Li et al. reported that overexpression of HAS2 in mesenchymal cells resulted in severe lung fibrosis and increased mortality in mice. In addition, in fibroblasts derived from idiopathic pulmonary fibrosis patients, the overexpression of HAS2 correlated with their ability to invade matrigel (61). By depleting HAS2 with siRNA in murine mesenchymal cells, cellular senescence could be induced *in vivo* in fibrotic fibroblasts in mice (58). In contrast to the previously reported beneficial effects of HMW-HA, these findings suggest that increasing the amount of HMW-HA synthesized by HAS2 acts as an important signal to induce pulmonary fibrosis. Moreover, Li et al. argued that downregulation of HAS2 could eventually be used to therapeutically induce cellular senescence in fibrotic tissues.

If a similar approach of induced cellular senescence can be employed as a conceivable approach to target tumor cells could not be shown so far.

## HA and Cancer

For humans, several studies have reported a key role of HA in tumorigenesis and various forms of epithelial and connective tissue cancers are associated with high levels of HA (62). On the one hand, an increased HA content has been shown for corporal fluids such as the urine of patients with bladder carcinomas (63, 64), the serum of patients with breast cancer (65), the saliva of patients with head and neck cancer (66), and the tumor interstitial fluid of colorectal cancers (67). On the other hand, HA levels can also be increased within the tumor either in the tumor parenchyma or the tumor stroma [reviewed in Ref. (68)]. As HA production by stromal cells can be stimulated by tumor cell-mediated signaling (69), HA is more frequently enriched in the stroma surrounding tumors than in the tumor parenchyma (70). For example, high stromal HA levels were found in patients with breast (71, 72) and ovarian carcinomas (73, 74) as well as in patients suffering from lung (75), brain (76), and prostate cancer (77). Nevertheless, malignant cancer cells themselves can also be responsible for an increased HA deposition (78, 79). For example, malignancy in lung (75), gastric (80), and colorectal cancers (81) is linked to the level of HA in the parenchyma. Thus, a significant number of studies showed that in cancer patients, HA concentrations are usually higher in tumors than in the surrounding healthy tissues (82). The extent of HA accumulation in both the tumor parenchyma and the tumor stroma can be correlated with the aggressiveness of cancers as elevated HA levels were known to stimulate processes involved in malignant growth such as cell proliferation, invasion, and metastasis (83). Therefore, an enhanced HA deposition, which is often accompanied by changes in the polymer size of HA, can be regarded as a reliable predictor for malignancy (68).

Malignant growth involves significant changes in the properties of ECM components leading to the establishment of a tumorigenic microenvironment supporting tumor cell survival, growth, invasion, and metastasis (84–86). Due to its important role as an ECM structuring molecule, HA is considered as an active participant in cancer-promoting processes especially those stimulating metastasis. Studies have shown that the metastatic potential of carcinoma cells is linked to the formation of pericellular HA matrices coating these aggressive tumor cells (87). The autocrine formation of these pericellular HA coats by invasive cancer cells themselves facilitates important steps in the metastatic cascade, such as tumor cell adhesion and extravasation (68). Therefore, it is proposed that metastatic tumor cells must acquire the ability to produce, assemble, and process their own portable HA-rich microenvironments in an autonomous manner in order to invade the circulation and to metastasize to ectopic compartments (88). This model that HA pericellular matrices function as portable microenvironments providing supply, nutrition, and protection for migrating cancer cells has been extensively reviewed and elaborated by Turley et al. (88).

The main reasons leading to an elevated HA deposition in various malignancies include alterations in the HA metabolism (89, 90). Although the mechanisms of HA accumulation can vary, changes in HA synthesis and/or degradation are most frequently observed in pathological processes. Therefore, it is important to understand how physiological HA concentrations *in vivo* are maintained.

## Rise and Fall—The Synthesis of HA

Hyaluronan is synthesized by hyaluronan synthases (HAS) (91, 92). There are three known human HA synthases that are numbered in the order of their discovery, and all are members of the HA synthases class I (93). The class I HA synthases contain a core of four transmembrane helices connected by at least one extended loop that carries the consensus sequence of the processive glycosyltransferases (94).

**Figure 4:**
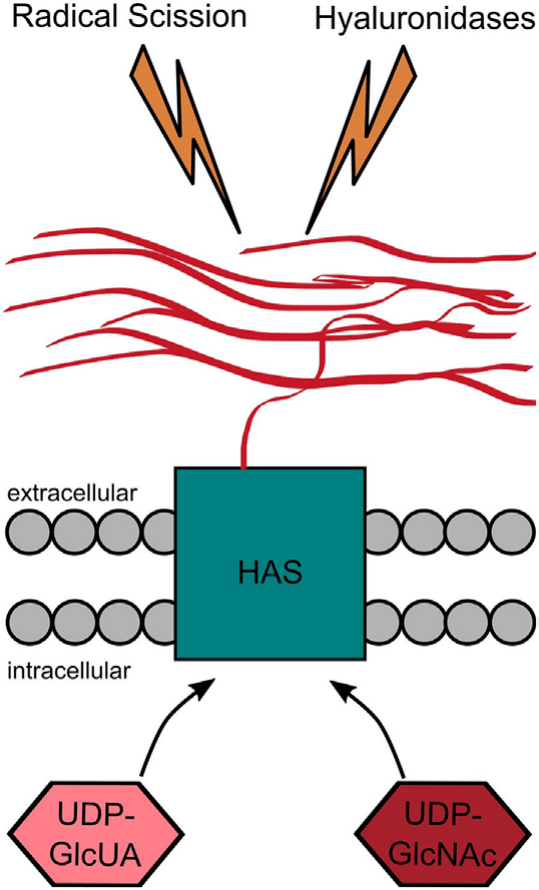
Hyaluronan (HA) metabolism. HA is produced by the HA synthases that catalyze the alternating addition of the uridine diphosphate (UDP)-activated monosaccharides (UDP-GlcUA and UDP-GlcNAc) to the reducing end of the growing HA chain. While the monosaccharides are added at the cytoplasmic site, the HA chain is simultaneously extruded to the extracellular space. HA is degraded either by radical scission or by enzymatic degradation. The latter one can either happen within the tissue itself or after drainage to the lymphatic system at different places within the body.

The HA synthases combine several functions that ultimately lead to the synthesis and translocation of HA to the extracellular space. They bind both uridine diphosphate (UDP)-activated monosaccharides and catalyze their alternating attachment to the reducing end of the growing HA molecule (Figure 4). This glycosyltransferase reaction occurs at the inner cell membrane and is directly linked to the extrusion of the polymer through the membrane spanning channel formed by the HAS or its dimerized form (94).

The three human HAS share a structural similarity of 55–70%, but they still differ in terms of their ability to synthesize HA (21, 95), their subcellular localization, enzymatic activity, and regulation (96, 97). When comparing the three HAS isoenzymes, HAS3 is the most active one, forming not only high but also low molecular weight HA (LMW-HA) (90, 98). HAS1 is able to produce high and LMW-HA such as HAS3, but is the least active of the three under normal conditions (90, 98, 99), whereas it is upregulated in states that are associated with inflammation (96). However, HAS2 seems to be even more important as it produces the HMW form of HA and is likely to be the HAS enzyme that is responsible for stress-induced increases in synthesis as it is found, for example, in shock, septicemia, inflammation, heavy wounding, and burn patients.

Furthermore, the deletion of the HAS2 gene leads to death already at early embryonic stages (98), the synthesis of HAS2 can be greatly influenced by external stimulants (97) and in some tissues, HAS2 is even expressed exclusively (100). Thus, the promoter area of HAS2 is most actively studied for responsive elements that bind regulatory transcription factors (97).

### HAS2—A Highly Regulated Enzyme

In general, the formation of the HA chain requires a high amount of energy as the formation of one disaccharide unit needs five ATP equivalents, two NAD cofactors, and one acetylCoA group as well as the compounds for the glucose and the glucosamine monosaccharide (93). Therefore, it is necessary for the cell to regulate HAS2 very tightly. The importance of HAS2 for the cell is emphasized by its various regulation and balancing mechanisms throughout transcription and translation of the enzyme as well as on a posttranslational level (97, 101, 102).

#### Regulation on DNA and mRNA Level

The synthesis of HA can already be influenced on the level of the expression of HAS2 as shown exemplarily above in the context of aging. The expression of HAS2 can be up- and downregulated by various transcriptional signals. Some, like the all-trans retinoic acid, which is a major developmental signal, act through their own nuclear receptor (103), while other signals are mediated either by phosphatidylinositol 3 kinase or G-protein coupled receptors. In cultured endothelial cells, the HAS2 transcription was also induced *via* nuclear factor κB (NF-κB) through tumor necrosis factor alpha and interleukin 1 beta (IL-1β) (104, 105). Likewise, an increased concentration of UDP-GlcNAc decreased the expression of HAS2 through the accumulation of two suppressive transcription factors (YYP and SP1) as a consequence of the elevated sugar levels [(106); reviewed in Ref. (97)].

Hyaluronan synthesis can also be regulated on mRNA level. There exists a natural antisense transcript of HAS2 (HAS-AS1). The exon 1 of HAS2-AS1 is complemental to the exon 1 of the HAS2 mRNA. By forming a duplex with the HAS2 mRNA, HAS2-AS1 stabilizes the HAS mRNA leading to an accumulation of HAS2 (101).

#### Posttranslational Modifications

There are several posttranslational modifications that can occur at different sites of the HAS2 and either decrease or increase the enzymatic activity (102). The glycosylation of serine 221 with an O-linked GlcNAc has been shown to increase the membrane stability of the protein and, thus, to prolong its half-life and to increase the HA synthesis (107). By contrast, phosphorylation of the HA synthases at varying sites might cause different effects (97). For example, phosphorylation with ERK increased the activity of all three HA synthases (101), while the phosphorylation of HAS2, induced by energetic stress, led to a reduced HA synthesis (97).

The direct link of HA synthesis to the metabolic state of the cell is adenosine monophosphate kinase (AMPK). AMPK has a special role within the cells as a metabolic sensor and regulator (108). After the activation of AMPK, the activity of HAS2 is dramatically inhibited due to the phosphorylation of the conserved threonine 110 in the cytoplasmic loop of HAS2 (108). However, AMPK does not interfere with the synthesis of HA synthases as the mRNA levels of the HA synthases were not altered by AMPK nor were the other HA synthases affected by AMPK (108).

The HAS2 activity can also be modulated by the ubiquitination at lysine 190 (102, 109) since the enzymatic activity was lost after a site-directed mutation of lysine 190 to arginine. These data suggest that HAS2 requires monoubiquitination for its activity. Furthermore, HAS2 was no longer able to form dimers after mutation (97).

#### Influence of Sugars

While GAGs are usually synthesized within the Golgi, where sugar concentrations are maintained at a constant and high level, HA is synthesized at the cell membrane and, therefore, susceptible to changes in cytoplasmic sugar concentrations (102).

Although UDP-GlcUA has a higher affinity to HAS2 than UDP-GlcNAc (93), UDP-GlcNAc was previously not considered to be a limiting factor in HA synthesis as it is more abundant in the cell. However, as the *K_m_* of UDP-GlcNAc is higher for all HAS enzymes, it is also possible to control the synthesis rate of HA through UDP-GlcNAc, so both sugars do have a direct influence on HA synthesis (95, 97).

The abundance of the sugars generated by an overexpression of enzymes in the anabolic pathway of the UDP-GlcUA led to increased HA accumulation (102), whereas the depletion of the precursor sugars either caused by toxins such as 4-methylumbelliferone (110–113) or induced by mannose (114) is able to specifically inhibit HA synthesis (97).

## Rise and Fall—The Degradation of HA

The HA turnover is surprisingly rapid in most tissues (see Figure 4). The HA half-life times range from a few hours to days in most of the body (115). While the synthesis takes place locally in the tissue (115), the degradation happens at different places. About 30% of the HA are turned over locally, whereas the remaining 70% enters the lymphatic drainage (116). Of those 70%, about 90% are removed within the lymphatic nodes (116). The HA-binding receptor in the lymphatic vessels and the lymph nodes is the lymphatic vessel endothelial hyaluronic acid receptor 1 (Lyve-1). It binds HA with high affinity, subsequently leading to the uptake of HA into the lymphatic vessels (116, 117). The endothelial cells in the liver (118), kidney, and spleen (19, 116) take up most of the remaining HA. The uptake of HA *via* clathrin-coated pits of the liver endothelial cells is inhibited if the hyaluronan receptor for endocytosis (HARE) is blocked (119). So far, this is the only case in which a knockout of a HA receptor leads to elevated HA levels in mice (116, 120). A final HA turnover route is provided by the excretion of HA from blood *via* urine. However, only 1% of HA is excreted through this glomerular filter with a cutoff of about 12 kDa (116, 121).

### Enzymatic Degradation of HA

Hyaluronan can either be degraded enzymatically or through radical scission. In eukaryotes, the HA degrading enzymes, also termed hyaluronidases (Hyals), are hydrolases and they are functionally active in a large pH range (122). So far, there are six Hyals known in humans: Hyal 1–4, HyalP, and PH20, which are all β,1-4 endoglucosaminidases (100).

Hyals can be characterized according to their pH-dependent activity. The acidic Hyals are active between pH 3 and 4. The human liver and serum Hyals (1–4) belong to this group. By contrast, the neutral Hyals are active at pH 5–8 containing PH20 and several venoms, such as snake and bee venom (123).

Hyaluronidase 1 (Hyal 1) is broadly distributed within the human body (124, 125). It is located in the lysosome and degrades the HA chain in concerted action with exoglycosidases to monosaccharides. Mutations of the enzyme are associated with lysosomal storage diseases, such as mucopolysaccharidosis type IX or hyaluronidase deficiency (100, 126–128).

Hyaluronidase 2 (Hyal 2) is a GPI-anchored receptor that operates in an acidic microenvironment at the cell surface (129, 130). It only hydrolyzes HMW-HA into LMW-HA (~20 kDa) (131) which is further hydrolyzed to oligomeric HA (oligo-HA) by Hyal 1.

In tumorigenesis, Hyal 1 and Hyal 2 act as a two-edged sword. A large amount of contradictory data exists regarding the exact role which the two Hyals possess in tumor progression. For example, Bouga et al. showed an increased expression of Hyal 1 and Hyal 2 in colorectal cancer (132). The overexpression of Hyal 1 also promoted mammary tumor growth and an increased tumor angiogenesis (133). Due to its high expression in the serum of epithelial ovarian cancers (134) and the urine of bladder cancer (135), Hyal 1 is also considered to function as a biomarker for those tumor types. However, a large amount of studies exist that contradict the concept of Hyals functioning as tumor promoters. For example, the overexpression of Hyal 1 inhibited tumorigenesis in rat colon cancer cells (136), while adenovirus-mediated expression of Hyal 2 could suppress tumor growth in mice (116, 137). Consistent with those observations, Frost et al. reported that a decreased Hyal 1 activity enhanced tumorigenesis in tobacco-related carcinoma of the head and neck region (138). The controversial roles of Hyal 1 and Hyal 2 in tumorigenesis are extensively reviewed elsewhere (5, 139–141). However, this selection of contradictory data already indicates that Hyal 1 and Hyal 2 might promote as well as suppress tumor growth and progression *in vivo* and that the regulation of Hyal 1 and Hyal 2 activity might be part of a tightly balanced regulation system involving synthesis and degradation pathways.

So far, there is no role described for the other four Hyals within tumorigenesis. Hyal 3 seems to have a non-enzymatic role regulating Hyal 1 (142); PH20 is a testicular enzyme that is important during mammalian fertilization events as it enables conception (143); Hyal 4 appears to be a chondroitinase without activity against HA (124, 144) and the pseudogene PHYAL 1 is transcribed but not translated in humans (144), however, it might be able to influence mRNA stability for homologous coding genes as pseudogenes are generally able to do so (145).

Regarding the mechanism of HA degradation on the tissue level, one can say that it involves Hyal 2 which gathers HMW-HA to the cellular surface, potentially in combination with cellular HA receptors such as CD44 (144, 146). The influence of CD44 in this process was underlined by using antibodies that block the clustering of CD44, which successfully inhibited the endocytosis and cleavage of HA dependent on the experimentally used cell type by at least 50% (116). By Hyal 2 HMW-HA is cleaved to 20 kDa fragments that are internalized by receptor-mediated endocytosis. Then, the HA fragments are intracellularly delivered to the endosome and subsequently to the lysosome where Hyal 1 in combination with two lysosomal β-exoglycosidases (β-glucuronidase and β-*N*-acetyl-glucosaminidase), finally, degrades the 20 kDa fragments. Within this process, there might be one step missing, in which oligo-HA is trimmed to a size small enough to exit the lysosome either by passive diffusion or by receptor-mediated exit (144). This scheme of divided responsibilities between Hyal 1 and Hyal 2 is supported by gene knockout studies. While Hyal 2 knockout is lethal in mice at the embryonic state or does have severe defects, the knockout of Hyal 1 can largely be compensated by the lysosomal β-exoglycosidases (21). A general cleavage mechanism for HA by Hyals was proposed and described earlier in further detail (122).

### Degradation of HA by Radicals

In addition to the enzyme-mediated cleavage, HA may also be degraded by radical scission caused by ROS or free radicals (see Figure 4) (130, 147). Interestingly, the radical scission of HA leads to chemically modified HA fragments containing chloramides and unsaturated end groups (148). These modified end groups might have different bioactivities compared to the fragments produced by Hyals.

The ROS are accumulated at the site of tissue injury, at sites of inflammation, and within the tumor microenvironment. They may provide a mechanism for generating HA fragments *in vivo* and may further exaggerate the inflammatory state as HA fragments have shown the significance of HA size in the course of disease (100).

## Size Matters—HA at Different Sizes

Obviously, a paradox exists between the high HA levels found in the naked mole rat, which are attributed to the animal’s cancer resistance, and the high HA levels in human cancerous tissues, which are indicative of a bad prognosis. To understand this fundamental issue, one must consider the different sizes in which HA can occur in the human body and their biological role.

**Figure 5:**
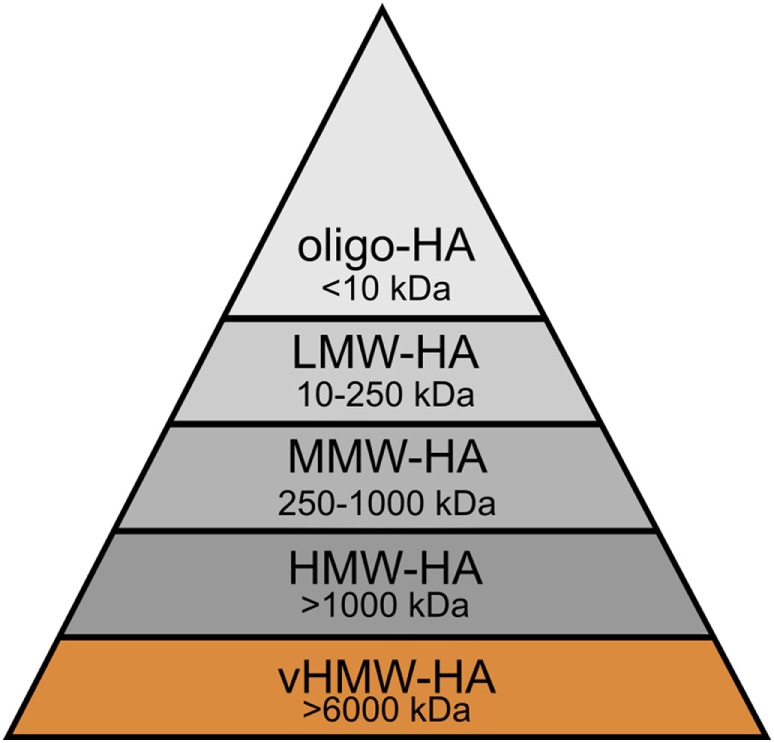
Molecular weight groups of hyaluronan (HA) in humans and the naked mole rat. HA can be distinguished into several groups according to its molecular weight: very high molecular weight HA (vHMW-HA), high molecular weight HA (HMW-HA), medium molecular weight HA (MMW-HA), low molecular weight HA (LMW-HA), and oligomeric HA (oligo-HA). vHMW-HA is unique and so far, only found in the naked mole rat.

In physiological as well as pathological conditions, the rapid HA turnover results in the constant presence of distinct forms of HA, each of which is characterized by polymer length and, thus, molecular weight. To understand the role of HA size in homeostasis and cancer, it is essential to distinguish these various molecular weight forms of high molecular weight HA (HMW-HA, >1,000 kDa), medium molecular weight HA (MMW-HA, 250–1,000 kDa), low molecular weight HA (LMW-HA, 10–250 kDa), and oligomeric HA (oligo-HA, <10 kDa) (5), and to compare them to the unique very high molecular weight HA (vHMW-HA, >6,000 kDa) of the naked mole rat (30) (Figure 5). By no means, these groups are distinctly distributed; in many settings, including cancer, the molecular weight of HA shows a polydisperse distribution in human tissues (149, 150).

### Caveats to HA Size

The number of studies attributing the functional diversity of HA to its size and molecular weight is comprehensive. Nevertheless, other variables such as conformation, content, and purity of HA as well as the content of HA in the ECM can also influence and affect the different roles of the GAG. For example, HA chains at different length scales can adopt different conformational states—extended, condensed, and relaxed—which are dependent upon pH, temperature, and salt concentration (151). Small changes in the local environment readily alter the conformation of HA and this was shown to affect the role of the GAG in biological processes such as the interaction with the complement system (152–154). Furthermore, the different preferences of short and long HA chains to involve in intermolecular interactions to form aggregates further expand the variety of molecular shapes of HA (155). Conformational change and/or self-association of HA shows influence on the viscoelastic (149) as well as binding properties (156) of HA. There is evidence that binding of HA to different proteins strengthens distinct conformations of the GAG resulting in complexes with unique architecture and biologic properties. In this context, the covalent modification of HA by heavy chains derived from the plasma inter-α-inhibitor serves as an example how the function of HA can be regulated *via* direct and indirect interaction of proteins (157). Artificial, chemical modification can also affect the function of HA *in vitro*: selectively *N*-acylated and *N*-butyrylated HA fragments modulated the production of inflammatory cytokines in human macrophages (158). The artificial modification of HA with sulfate groups influenced the binding affinities to recombinant human bone morphogenetic protein-4 (159) and recombinant human transforming growth factor-β1 (TGF-β1) (160). In both cases, a higher degree of sulfation led to a stronger interaction (159, 160). Sulfated HA was demonstrated to inhibit Hyals and to function as a molecule with antitumor activity (161).

Another issue to consider when investigating the biological activity of HA is the source and way of preparation. Concerns have arisen that the pro-inflammatory effects reported for LMW-HA and oligo-HA are the result of inadequate purification and processing. HA from the umbilical cord was associated with DNA and protein contaminants (162), which possess pro-inflammatory activity (163). Dong et al. also reported that endotoxin contamination in human umbilical cord HA, bovine testes, and *Streptomyces hyaluronlyticus* hyaluronidase preparations was responsible for cytokine production in dendritic cells or macrophages. By contrast, endotoxin-free pharmaceutical grade HA and HA fragments failed to induce similar inflammatory responses (164). Such results emphasize the high importance of excluding contamination in HA preparation.

It is important to bear in mind that the functional diversity of HA can partially be attributed to factors, such as conformation, content, modification, and purity. However, standing alone, none of them can fully explain the ambivalent roles of HA in the ECM. Closer assessment reveals that the size of HA directly interlinks with the varying biological activities of the GAG and, thus, functions as key player and main contributor. For this reason, this review specially emphasizes the importance of the distinct molecular weight forms of HA which can be found in humans and naked mole rats (Figure 5).

### High Molecular Weight HA

The molecular weight of HA varies and has great impact on the physiological functions and activities of HA (27): in homeostasis, HA is found in its HMW form in almost all human tissues (5). Due to its biophysical properties, HMW-HA serves as lubricant, space-filler, and shock absorber in joints and connective tissues (5, 29). HMW-HA also plays an essential role during embryogenesis (18, 21). Embryonic stem cells highly express the polymer throughout the whole process of epithelial–mesenchymal transition (165). The presence of endogenously produced HMW-HA-rich matrices is critical in the development of various tissues, such as the brain (166), the hematopoietic system (167), and the heart (168).

Furthermore, HMW-HA promotes anti-inflammatory, anti-proliferative, and anti-angiogenic effects (5, 27, 28). For example, intraperitoneal treatment with HMW-HA completely inhibited monocyte and neutrophil infiltration in a lipopolysaccharide-induced lung injury model (169). HMW-HA deposition is also reported to have a favorable outcome in wound healing [reviewed in Ref. (170)].

The protective roles of HMW-HA in the human body do not only apply to inflammation, embryogenesis, and wound healing but are also visible in tumorigenesis. In different tumor models, HMW-HA prevented cancer cell migration (171) and regrowth (172), as well as the synthesis of pro-inflammatory mediators (173). The protective effects of HMW-HA are not solely limited to primary tumor progression. A recent study also indicated an antimetastatic role for HMW-HA (174). Treatment with HMW-HA strengthened the monolayer integrity of cancer lymphatic endothelial cells, thus, preventing cancer cell outgrowth (174). Due to its anticancer effects observed after exogenous application, HMW-HA is regarded as an attractive agent to support both adjuvant and neoadjuvant chemotherapy (5, 83).

Opposing to the cancer resistance of the naked mole rat due to vHMW-HA and the previously described involvement of HMW-HA in cancer inhibition, cancer-promoting effects of HMW-HA have also been reported. For instance, HMW-HA is capable of promoting angiogenesis and cell migration in the hepatocellular carcinoma cell line HepG2iso and in primary human umbilical vein endothelial cells *via* CXCL12-dependent signaling through the HA receptor CD44. In contrast to that, small HA oligosaccharides inhibit these effects (175). Guo et al. showed that not only angiogenesis but also tumor lymphangiogenesis is promoted by HMW-HA. Xenografts with hepatocellular carcinoma Hca-F cells were used to observe the effects of HA on lymphangiogenesis. As a result, HMW-HA treated tumors exhibited intratumoral lymphatic vessels that could not be detected in untreated tumors (176).

Moreover, HMW-HA plays a role in several aspects regarding breast cancer. Bourguignon et al. reported that binding of HMW-HA to CD44 promotes chemotherapy resistance and anti-apoptosis in breast cancer cells. These oncogenic effects are caused by an activation of protein kinase C ε (PKCε) and a subsequent microRNA-21 production *via* Nanog signaling (177). HA/CD44 signaling is also involved in the invasive behavior of breast cancer cells. Binding of HA polymers to CD44 activates the c-Src kinase that leads to microRNA-10b production *via* Twist phosphorylation. Eventually, these occurrences enable invasion of breast tumor cells due to downregulation of HOXD10, a tumor suppressor protein, the overexpression of RhoC, and activation of ROK (178).

Stromal fibroblasts in the microenvironment of lung tumors presented tumor-promoting features, including tumor growth, survival, and drug resistance. The p38-HA pathway was identified as crucial regulator of these tumor-promoting fibroblasts. Kras-driven lung cancerogenesis leads to the activation of p38MAPK which subsequently supports the activation of HAS2. As a complete knock-down of p38MAPK is lethal in the embryonic state, a knock-in mouse strain with a substitution of Tyr182 with Phe was created (p38^ki/ki^ mice). The substitution causes a significant decrease of p38MAPK expression without lethal consequences. The expression of HAS2 is downregulated in lung fibroblasts from p38^ki/ki^ mice. Co-culture of lung cancer cells with lung fibroblasts of p38^+/+^ or p38^ki/ki^ mice revealed a decreased tumor cell growth when cultivated with p38^ki/ki^ cells. Interestingly, the addition of HMW-HA to the cells completely reversed the lacking tumor-promoting effects of p38^ki/ki^ cells. The same results were obtained by an overexpression of HAS2 in p38^ki/ki^ cells due to a HAS2 plasmid. This demonstrates that the p38MAPK-dependent activation of HAS2 and the subsequent production of HMW-HA are crucial for the tumor-promoting features of fibroblasts (179).

The variety of studies reveals that the effects of HMW-HA on different aspects of cancer, such as development, invasiveness, or drug resistance, are opposing and seem to greatly depend on cancer type.

Nevertheless, there are several studies reporting the important role of HMW-HA as a tissue protector and homeostasis promoter after injury and inflammation (5, 83, 180). However, as we have particularly seen for cancer, diseases are associated with an increased HA level. Regarding the protective effects of HMW-HA, the question arises which roles the other molecular weight groups of HA play (see Figure 5).

Pathological circumstances, such as inflammation, show evidence for an elevated HA fragmentation resulting in a higher level of HA polymers with a lower molecular weight [reviewed in Ref. (29)]. Especially in human cancer, the weight distribution of HA is shifted toward lower molecular weight forms. These shorter bioactive HA fragments can interact with cancer cells and influence their behavior differently compared to HMW-HA (5, 181–183).

In cancer and other diseases, the harmful effects of the lower molecular weight forms of HA—oligo-HA in particular—predominate.

### Oligo-HA

The equivocal effects of oligo-HA either acting as tumor promoter or tumor suppressor represent a disputed issue within the scientific community. One of the main fundamental obstacles to clarifying this issue lies in the fact that the effects of oligo-HA on transformed cells are much more pleiotropic than on the non-transformed counterparts affected in other diseases (5).

While HMW-HA is known to support tissue homeostasis, HA breakdown products can be regarded as a cellular alarm signal (29, 139). There are several pieces of experimental evidence for the pro-inflammatory effects of oligo-HA enhancing and promoting tumor growth and metastasis: oligo-HA stimulated the proliferation of papillary thyroid carcinoma cells *in vivo via* a toll-like receptor (TLR) 4-dependent signaling mechanism (182). Further involvement of TLR4 as a mediator of tumor-promoting oligo-HA signaling was reported for a melanoma tumor model. Oligo-HA exposure of human melanoma cells led to the activation of the NF-κB pathway resulting in an increased expression of matrix metalloproteinase (MMP) 2 and the inflammatory cytokine IL-8 (184). Another study reported an oligo-HA-induced physical interaction between the main HA receptor CD44 with TLR2 and TLR4 causing pro-inflammatory cytokine and chemokine production in breast cancer cells *via* NF-κB transcription (181). HMW-HA could not activate this pro-inflammatory signaling cascade (181). Likewise, only oligo-HA, not HMW-HA, increased the phosphorylation of the receptor tyrosine kinase c-Met, also known as hepatocyte growth factor (HGF) receptor, in chondrosarcoma cells resulting in an enhanced cell proliferation, differentiation, and invasion (185).

Oligo-HA also promotes early steps in metastasis. For example, a recent study showed that oligo-HA disrupted tight junctions in a cancer lymphatic endothelial cell monolayer and promoted cancer lymphatic metastasis by weakening cellular integrity (174). It seems likely that oligo-HA exerts similar effects on non-transformed lymphatic vessel cells since the amount of oligo-HA in the tumor interstitial fluid of colorectal cancers could be correlated with lymphatic invasion and lymph node metastasis (67).

Furthermore, oligo-HA acts as a potent inducer of angiogenesis (186). HA fragments mediate their angiogenic properties either by directly activating endothelial cell differentiation (187) or by stimulating the secretion of angiogenic growth factors (188). In response to oligo-HA, both tumor cells and tumor-associated stromal cells, such as fibroblasts and macrophages, can synthesize angiogenic factors known to affect endothelial cell proliferation, migration, and differentiation (189). The role of the immune system in oligo-HA-mediated angiogenesis was recently reviewed by Spinelli et al. (190). It is suggested that oligo-HA modulates angiogenesis through the activation of Raf-1, ERK1/2, and early response genes, including c-fos and c-jun through the receptors CD44 and the receptor for HA-mediated motility (RHAMM) (139, 191). Interestingly, there are reports that the angiogenic potential of oligo-HA depends on the exact size of the oligomer (192). In this context, Stern et al. provides an overview of signal transduction pathways addressed by HA oligomers with different polysaccharide lengths (29). This indicates that HA size is a main factor for deciding which cellular responses are addressed and to which extent they are stimulated. Nevertheless, oligo-HA-mediated angiogenesis serves as an example of how malignancies can exploit normal physiological functions, originally attributed to healing processes, for their own purposes (139). Therefore, with the help of oligo-HA, different tumor cells can promote their adhesion, angiogenesis, and invasion by manipulating cellular pathways.

### Oligo-HA—Only the Bad Guy?

In contrast to its tumor-promoting effects, oligo-HA has also shown protective effects in cancer. For example, Zeng et al. observed the inhibition of B16F10 melanoma growth *in vivo* after the injection of oligo-HA (193). One of the main mechanisms by which oligo-HA was found to mediate its tumor-suppressing effects is the activation of apoptosis.

The administration of oligo-HA triggered apoptosis in many types of tumors (194, 195), whereas healthy cells were left unaffected (195). Several studies reported that the specific activation of apoptosis in tumor cells depends on the interaction of oligo-HA with CD44. For example, oligo-HA suppressed tumor progression in a highly metastatic breast cancer cell line as it disrupted the endogenous interaction of HMW-HA with CD44 (183).

However, oligo-HA-mediated signaling does not only influence tumor cells themselves. In a colorectal carcinoma model, oligo-HA triggered the activation of the immune system by enhancing the expression of costimulatory molecules on dendritic cells (196). Therefore, oligo-HA can exert its anticancer activity not only by inducing apoptosis but also by enhancing the body’s immune response (196).

Furthermore, oligo-HA may offer a novel basis for the development of anticancer drugs as the exogenous application of oligo-HA converted chemoresistant tumor cells into drug-sensitive cells (195). For example, it was shown that oligo-HA can sensitize various tumor cell lines such as lymphoma cells (197), ovarian carcinoma cells (198, 199), and myeloid leukemia cells (200) to chemotherapy.

There is no doubt that oligo-HA is a very interesting molecular weight form of HA. However, by looking at an ever-increasing number of functionally and pathologically distinct tumors, further research is required to uncover the main underlying issues for the shape-shifting properties of this molecule. Therefore, it is most likely that the exact role of oligo-HA in a specific tumor setting varies depending on the oligomer size, concentration, and modification, as well as the cancer type and the involved healthy tissue.

### HA Size As Sensor Element

**Figure 6:**
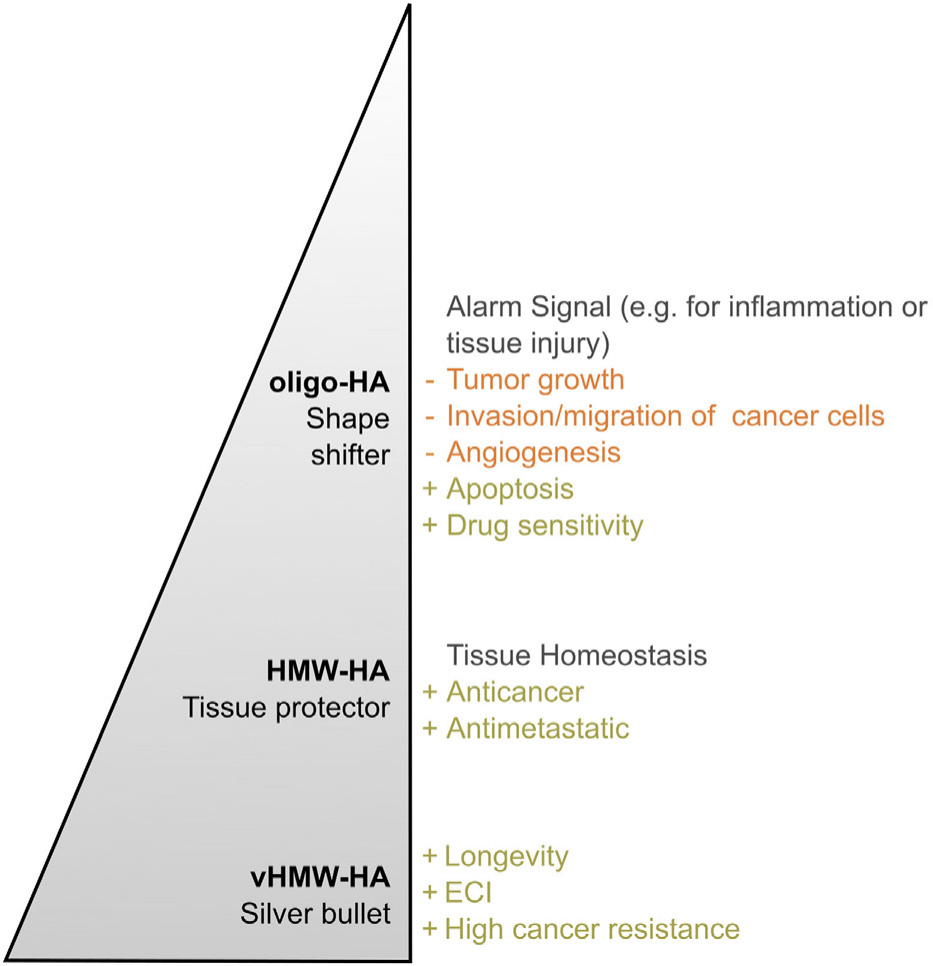
Hyaluronan (HA)—a polymer with a multitude of roles. The effects of HA in both the healthy and diseased organism change with the molecular weight of the polymer *in vivo*. This becomes particularly obvious by comparing very high molecular weight HA (vHMW-HA) found in naked mole rats with the most important human molecular weight forms, namely high molecular weight HA (HMW-HA) and oligomeric HA (oligo-HA). Especially in the process of tumorigenesis, each molecular weight group can be described with its own characteristic role. While oligo-HA shows shape-shifting effects in cancer, HMW-HA mainly protects the human tissue. As reviewed above, various studies report this role distribution for HMW-HA and oligo-HA. Nevertheless, role reversal seems to be possible. vHMW-HA represents a silver bullet unique for naked mole rats as the high cancer resistance of the animal was attributed to this molecular weight form. The pyramid shape symbolizes the polymer length and, thus, molecular weight of each HA group.

In conclusion, HMW-HA and oligo-HA can trigger different cellular functions and responses (Figure 6). In the homeostatic state, HMW-HA represents the most abundant molecular weight group of HA, whereas under pathological circumstances, such as inflammation and cancer, the size distribution shifts, and smaller HA polymers become present at significant levels (29). Smaller HA polymers bind HA receptors with the same affinity as HMW-HA but with reduced avidity due to fewer multivalent interactions (201). However, the length of HA influences the number of HA receptors bound by a single HA molecule. Thus, the induced HA receptor clustering depends on the molecular weight of HA (202, 203). In the case of HA oligosaccharides, this becomes visible in the physiological as well as the pathological setting. The constant fragmentation of HMW-HA is required to coordinate cellular activities, for example, in wound healing [reviewed in Ref. (170)] as well as in tumor progression. As oligo-HA and HMW-HA compete in receptor binding (5), the exact HA size distribution in a cellular microenvironment determines eventually which kind of cellular responses are addressed. Therefore, HA size matters (180) and, moreover, can be regarded as a sensitive element reflecting the state of a cellular microenvironment. In this context, the biology of HA was stated to function as a cellular biosensor system analyzing and conveying environmental states (149).

## Hyaladherins—Mediators of Cellular Response

As shown above, the effects mediated by HA are strongly dependent on its size in tissue homeostasis as well as in pathologies, such as cancer. However, in order to translate its size-dependent signals into cellular functions, HA needs to interact with the HA-binding proteins, the main mediators of HA-induced cellular response.

The HA-binding proteins, also known as hyaladherins, comprise specific motifs to bind HA, including the link module and the B(X_7_)B motif (16, 21). Some of those hyaladherins, such as versican and aggrecan, serve as a part of the ECM, others act as HA receptors directly interfering with the cellular functions (16, 21). The main HA receptors are CD44, __RHAMM, __Lyve-1, and __HARE (101, 204). Whereas HARE is responsible for the endocytosis-mediated clearance of GAGs, including HA, Lyve-1 is known to regulate the tissue HA level by mediating the transport of HA from tissues to the lymphatic system (101, 204). In contrast to Lyve-1, HARE, and CD44, RHAMM lacks a transmembrane domain and is, thus, localized intracellularly within the cytosol or the cell nucleus and can be secreted to the extracellular space (21, 101). Due to its ability to interact with the cytoskeleton as well as with signaling molecules, including diverse kinases (15, 101), RHAMM is a key player in regulating cell motility and migration and, thus, is especially involved in the processes of tissue injury and wound healing (204). Besides, RHAMM was also reviewed to regulate mitosis (21) as well as the proliferation of fibroblasts (204) and to be expressed on a variety of cell types, including endothelial but also tumor cells (101, 204).

Although the HA receptors are the most commonly known regulators of HA-dependent cellular responses, there are more HA-related proteins influencing the cell’s behavior: for instance, the hyaluronidase Hyal 2 is not only capable of binding and degrading HA as described above. Hyal 2 was also reported to act as a receptor for TGF-β1 by recruiting the tumor suppressors WW domain-containing oxidoreductase (WWOX) and Smad4 (205, 206). As a consequence, a Smad4/Hyal 2/WWOX signaling complex was shown to be formed and translocated to the nucleus where it increased the SMAD-promoter-dependent transcriptional activity and—in case of overexpression of the signaling complex—also led to apoptosis (205, 206). Further treatment of the cells with HA enhanced the formation of the signaling complex as well as its translocation (206), emphasizing an involvement of HA. The same signaling pathway may underlie the zinc finger-like protein that regulates apoptosis (Zfra)-induced tumor suppression and cancer resistance in mice (207) as well as neuronal death caused by traumatic brain injury in rats (206, 208). Whereas the Hyal 2/WWOX/Smad4 signaling pathway was shown to be CD44 independent (205), further Hyal 2-mediated functions, such as the HA degradation process [(146); described in Chapter 7.1] and the CD44–ezrin, radixin, moesin (ERM)-mediated cell motility (209), rely on Hyal 2 as a co-receptor with CD44 for HA.

Since the HA receptors and Hyal 2 have generally been reviewed extensively elsewhere (15, 21, 101, 204, 208), within this review, we will focus on the most abundant HA receptor: CD44.

### CD44—A Highly Diverse Cell Surface Receptor

CD44 is a type I transmembrane protein that consists of an N-terminal HA-binding domain, a membrane-proximal stem region, a transmembrane, and a cytoplasmic domain (from extracellular to intracellular) (210). Due to alternative splicing of the CD44 transcript, there exists a variety of CD44 isoforms differing mainly in the length of the membrane-proximal stem region (210). Whereas the most widely expressed standard CD44 (CD44s or CD44h) includes none of the variant exons, the variant CD44 isoforms (CD44v), containing some of the variant exons, are expressed in a more restricted manner. For example, CD44v can be found on epithelial, endothelial, and immune cells, but they are also associated with diverse diseases, such as rheumatoid arthritis, diabetes, multiple sclerosis, and cancer (211–213).

This variety of CD44 isoforms is even more increased by the differing posttranslational modifications of the CD44 molecule that include glycosylation of the extracellular domains, palmitoylation of the membrane-proximal intracellular part, phosphorylation of the cytoplasmic domain, as well as sulfation and the attachment to GAGs (211, 214). In particular, the posttranslational glycosylation of CD44 was shown to modulate the receptor’s HA-binding affinity (215). However, studies reported contradicting effects, indicating inhibitory as well as stimulatory effects of CD44 glycosylation on the HA-binding affinity (214, 216). These opposing effects might be due to the presence or absence of *N*-acetylneuraminic acid, also known as sialic acid, in the attached glycan as observed in a molecular simulation study investigating the HA-binding properties of the CD44 receptor (214). By contrast, the palmitoylation of CD44 is responsible for the receptor’s affinity to the so-called lipid rafts, specific membrane regions enriched in adhesion and signaling molecules (217). As a part of those signaling platforms, CD44 is able to associate with members of the Src kinase family and receptor tyrosine kinases modulating cell motility as well as signal transduction (211). Thus, the posttranslational modifications of CD44 not only regulate the HA-binding affinity but also the intracellular signaling and, thus, the cellular response to HA binding by CD44.

Although HA is known to be its principal ligand, CD44 was also shown to bind ECM proteins, such as collagen or fibronectin, as well as diverse growth factors, cytokines, chemokines, MMPs, and osteopontin (210, 211). Some of those ligands require specific posttranslational modifications or parts of the variant exons in order to bind CD44. For example, the fibroblast growth factor 2 (FGF2) binds to the heparin sulfate site on variant exon 3. HGF and vascular endothelial growth factor (VEGF) are bound through a site on CD44v6 (218). By binding such factors, CD44 might function as a gathering site bringing together enzymes and substrates as well as ligands and their receptors (210).

Regarding signal transduction, CD44 not only functions as a co-receptor for several pathways, such as the ERBB signaling [reviewed in Ref. (68, 210)]. The cytoplasmic domain of CD44 may even directly associate with diverse signaling molecules [reviewed in Ref. (68, 210)]. Furthermore, the cytoplasmic domain of CD44 provides an ankyrin-binding site as well as a motif to bind the ERM proteins. As those ERM proteins may also bind to filamentous actin, they serve as linker molecules between CD44 and the actin cytoskeleton, so that CD44 may impact the ERM-mediated signaling as well as the organization of the actin cytoskeleton (210, 218). However, this process seems to be tightly regulated by phosphorylation of the ERM proteins as well as merlin, a protein related to the ERM proteins but acting as their antagonist (210, 219). Whereas phosphorylated ERM proteins bind to CD44 and induce cell growth, dephosphorylated merlin replaces ERM proteins from their binding site mediating cell growth arrest (210, 219). In naked mole rats, this interplay between merlin and CD44 is crucial for the ECI as described above. Since the dephosphorylation of merlin might be induced by high cell density or an accumulation of HMW-HA in the extracellular microenvironment (210), the CD44-mediated signaling may act as a biosensor for the cell’s microenvironment.

**Figure 7:**
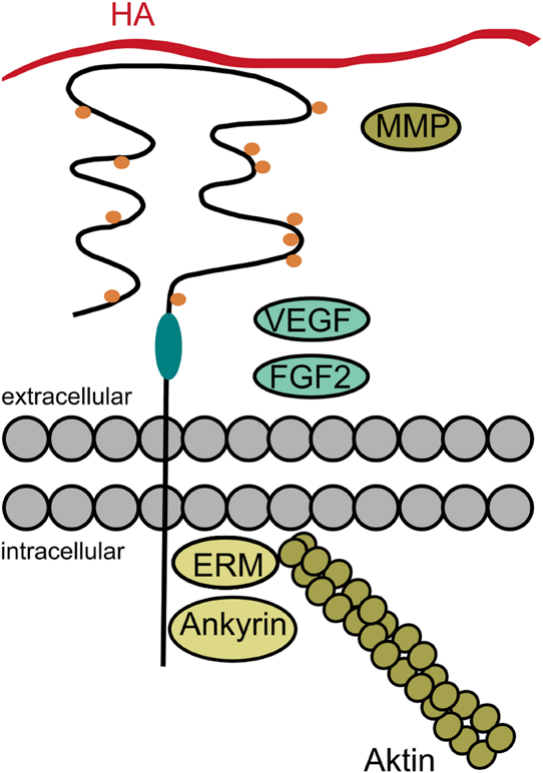
The diversity of CD44. Cellular responses induced by CD44 may be regulated on several levels: the alternative splicing in the stem region of CD44 (indicated in turquoise) may give rise to additional posttranslational modifications and binding sites. The posttranslational modifications (indicated as orange dots) may regulate the receptor’s binding affinity and localization. Besides its principal ligand hyaluronan (HA), CD44 and especially its variant isoforms may also bind other ligands, such as fibroblast growth factor 2 (FGF2), vascular endothelial growth factor (VEGF), and matrix metalloproteinases (MMPs). Intracellularly, CD44 bears binding sites for ankyrin and the ezrin, radixin, moesin (ERM) proteins in order to interact with the cytoskeleton. But CD44 may also associate with a variety of receptors and signaling molecules as a co-receptor or *via* its cytoplasmic domain (data not shown in the figure).

Taken together, the diversity of cellular responses induced by CD44 is regulated on several levels: (i) alternative splicing, (ii) posttranslational modifications, (iii) ligand binding, and (iv) association of CD44 with signaling as well as cytoskeletal molecules (Figure 7).

However, the diverse processes resulting from the variety of CD44 and its interaction partners [reviewed in Ref. (210–212, 218)] depend on the cellular microenvironment, the cell type, and the growth conditions (211). Thus, the CD44 functions are also exploited in diverse pathologies, such as cancer.

### CD44—Contributor to Malignancy

In human tumors, not only the expression level of CD44 is increased (220), for solid tumors, CD44 was also reported to be overexpressed in an activated, high-affinity state in tumor-derived compared to non-tumorigenic cells (211). Moreover, the expression pattern of the CD44 variants is altered in a broad range of human tumors [reviewed in Ref. (220)]. In tissues of human colorectal cancer, the expression level of CD44v6 was shown to be even correlated with tumor progression since the number of CD44v6-positive tumors as well as the number of positive cells and the expression level of the CD44 isoform within the tumors were increasing with advancing stages of the disease (221). Thus, CD44 and its variant isoforms might be associated with tumor-promoting processes.

Indeed, the expression of CD44 variants has an impact on tumor progression. For example, CD44v6 is able to bind ligands, such as HGF and VEGF (218), activate their receptors, c-Met and VEGFR-2, and recruit the F-actin-bound ERM proteins to its cytoplasmic domain as required for the c-Met- and VEGFR-2-induced intracellular signal transduction [(222–224); reviewed in Ref. (225)]. Through those signaling pathways, CD44v6 is related to processes, such as cell proliferation, differentiation, and migration (223) as well as angiogenesis (224), and processes associated with metastasis formation.

Furthermore, human tumor tissues of several entities, including gliomas, breast, lung, colon, and ovarian carcinomas, have shown an increased cleavage of CD44 within the extracellular domain (226). This proteolytic cleavage of CD44 is induced by extracellular Ca influx, PKC, as well as the Rac and Ras oncogenes, and mediated by MMPs as well as a disintegrin and metalloproteinase (ADAM) proteins. As a consequence, soluble CD44 is released to the ECM competing with the membrane-bound CD44 for HA binding and, thus, regulating cell adhesion to as well as cell migration on the pericellular HA coat (218, 227). Besides, the extracellular CD44 cleavage might also induce the presenilin-dependent γ-secretase-mediated proteolysis of the remaining CD44 within its transmembrane region giving rise to a separated CD44 intracellular domain that may trigger the transcription of the *CD44* gene to regenerate the expression of CD44 on the cell membrane (218, 227, 228). Thus, by modulating the CD44 turnover (227), the function of CD44 in cell adhesion and migration is exploited to mediate tumor cell migration.

However, these are only examples for the role of CD44 in cancer. As a consequence of its structural diversity and its ability to interact with a plethora of extracellular ligands, transmembrane proteins as well as cytoplasmic molecules, CD44, and its variants are generally involved in a high number of tumor-promoting processes [reviewed in Ref. (68, 210, 211, 218)]. Nevertheless, considering the naked mole rat, there was one process crucial for its cancer resistance: the contact inhibition.

The contact inhibition is a process that usually induces cell growth arrest in cells when they reach complete confluence and contact each other (38). In naked mole rats, this induction of cell growth arrest already takes place at lower cell densities than in other organisms due to their so-called ECI that is based on an interaction of the naked mole rat’s vHMW-HA with its receptor CD44 and a cytoplasmic association of CD44 to merlin (see Figure 3) (30). This HA-induced association between CD44 and merlin was also observed during contact inhibition in a rat schwannoma cell line among others (219). In this study, Morrison et al. even showed that the interaction of CD44 and merlin is highly regulated by a contact-induced hypophosphorylation of merlin resulting in the replacement of the cell growth-mediating ERM proteins by the hypophosphorylated merlin at the cytoplasmic domain of CD44 and the induction of cell growth arrest (219).

However, cancer cells lose their ability of contact-induced cell growth arrest and gain the function to grow in an anchorage-independent manner (38, 68). This anchorage-independent growth of cancer cells was reported to be enhanced by HA (68, 229, 230) and mediated by downstream activation of the phosphoinositide 3-kinase (PI3K)/Akt survival pathway (68). Especially, an overexpression of the HAS2 resulting in an overproduction of HA showed an increase in anchorage-independent growth (229, 230). But by overexpressing soluble CD44 or applying HA oligomers this process could be inhibited (194, 231). Ghatak et al. further showed that the inhibition of anchorage-independent growth by HA oligomer treatment coincides with an induction of apoptosis and a downregulation of the PI3K/Akt survival pathway (194). Similar effects were observed when treating the cancer cells with an antibody against CD44 (194). Thus, CD44 seems to be involved in the processes of contact inhibition in normal cells as well as the anchorage-independent growth of cancer cells. Since the latter could be inhibited by HA oligomer treatment, it also seems like the size of HA matters. However, the exact mechanisms causing the switch from contact-sensitive toward anchorage-independent growth in cancer cells remains to be elucidated.

## Epilogue

Overwhelmed by the sheer flood of faces the simple sugar chain of HA can assume, the researcher looked away from his laptop and out of the window. Surely, there were tremendous obstacles to be overcome in the future but for the first time in months he had an idea how to carry on his research to fight cancer. Thinking of this extraordinary rodent and the unexpected, though auspicious, connection to the sugar, all mammals bear within them, he smiled, grabbed his coat, and went off to the lab.

## Author Contributions

This review was written by LB, A-KH, EMS, SW, and FW within the GlycoScience seminar at the University of Heidelberg taught by HB. All authors participated in the development of the overall story line and actively contributed to all parts.

## Conflict of Interest Statement

The authors declare that the research was conducted in the absence of any commercial or financial relationships that could be construed as a potential conflict of interest.
